# Adaptation of the short intergenic spacers between co-directional genes to the Shine-Dalgarno motif among prokaryote genomes

**DOI:** 10.1186/1471-2164-10-537

**Published:** 2009-11-18

**Authors:** Albert Pallejà, Santiago García-Vallvé, Antoni Romeu

**Affiliations:** 1 Department of Biochemistry and Biotechnology, Rovira i Virgili University, Tarragona, Catalonia, Spain

## Abstract

**Background:**

In prokaryote genomes most of the co-directional genes are in close proximity. Even the coding sequence or the stop codon of a gene can overlap with the Shine-Dalgarno (SD) sequence of the downstream co-directional gene. In this paper we analyze how the presence of SD may influence the stop codon usage or the spacing lengths between co-directional genes.

**Results:**

The SD sequences for 530 prokaryote genomes have been predicted using computer calculations of the base-pairing free energy between translation initiation regions and the 16S rRNA 3' tail. Genomes with a large number of genes with the SD sequence concentrate this regulatory motif from 4 to 11 bps before the start codon. However, not all genes seem to have the SD sequence. Genes separated from 1 to 4 bps from a co-directional upstream gene show a high SD presence, though this regulatory signal is located towards the 3' end of the coding sequence of the upstream gene. Genes separated from 9 to 15 bps show the highest SD presence as they accommodate the SD sequence within an intergenic region. However, genes separated from around 5 to 8 bps have a lower percentage of SD presence and when the SD is present, the stop codon usage of the upstream gene changes to accommodate the overlap between the SD sequence and the stop codon.

**Conclusion:**

The SD presence makes the intergenic lengths from 5 to 8 bps less frequent and causes an adaptation of the stop codon usage. Our results introduce new elements to the discussion of which factors affect the intergenic lengths, which cannot be totally explained by the pressure to compact the prokaryote genomes.

## Background

Prokaryote genomes are considered compacted genomes with only small fractions of their genomic DNA assigned to intergenic regions [1]. The percentage of these non-coding regions varies across the prokaryote species, and does not depend on genome size or gene content, even though the latter variables strongly correlate [2]. The spacers between a pair of genes were divided into three types according to their transcriptional direction: i) unidirectional, ii) convergent and iii) divergent [1]. Here we use the co-directional term rather than the unidirectional one. These three types of spacers differ in the type of regulatory signals they contain. In prokaryotes, most of the co-directional genes are involved in operons [3,4]. The spacers between these genes may contain translational signals such as the Shine-Dalgarno (SD) sequence. The intergenic spacers between convergent genes may contain terminators for both genes while the divergent ones have only promoters and other upstream transcriptional signals. The different types of intergenic regions in prokaryotes, including the convergent and divergent ones (all of which are inter-operonic) and the co-directional ones (which are mainly intra-operonic), evolve under the same evolutionary pressures. Selection pressure [1] and deletional bias [2] have been proposed as the main forces responsible for minimizing the amount of non-functional DNA in prokaryote genomes. Deletion bias is the mechanism that shapes the prokaryote genomes, while selection pressure may establish an equilibrium with deletional bias in order to maintain minimally required amounts of non-coding DNA. These minimally required amounts of non-coding sequences are required to accommodate essential regulatory signals [1] and DNA replication sequences [5,6]. According to the genomic compactness, prokaryote genomes have intergenic distances that are much shorter than gene lengths and relatively shorter than those in eukaryote genomes [7]. Eukaryote genomes have a much wider range of genome sizes and contain protein-coding genes that are typically interrupted by introns and have longer intergenic regions.

One of the regulatory sequences affected by the short distances between prokaryote genes is the SD sequence. In 1974, Shine and Dalgarno found a sequence (5'-GGAGGU-3') at the 5' of the initiation codons in several messenger RNAs (mRNAs) of *Escherichia coli *that was complementary to the 3'-CCUCCA-5' sequence located at the tail of the 3'-end of the 16S ribosomal RNA (rRNA) [8]. It has been suggested that a strong SD sequence, though not mandatory in translation initiation, may compensate for a weak start codon and counteract mRNA secondary structures that hinder access to the start codon [9,10]. Although the genes with a SD sequence are widely found in prokaryote genomes, previous studies have also shown that there is a significantly and previously underestimated population of genes without a SD sequence [11-14]. Moreover, the exponential increase of the fully sequenced genomes has provided thousands of examples of leaderless genes or genes without a SD sequence in prokaryote genomes [15]. It has been suggested that the leaderless genes could use an independent pathway in their gene translation, while leader genes without a SD sequence must use alternative unknown mechanisms in their translation initiation [16,17].

Among the genes that have a SD motif, the ribosome does not need a perfect distance between the SD sequence and the start codon for the initiation of translation. However, when the SD sequence is located within four or as far as 13 nucleotides from the start codon, the gene expression decreases dramatically [18-20]. Therefore, there are apparently structural constraints that require an optimal space between the SD motif and the start codon. This sequence has mainly been found 7 to 12 nucleotides upstream of the start codon [12,21,22]. Taking this into account, the intergenic distances are an important feature of the prokaryote genomes that may correlate with the SD presence [12]. Many genes are sufficiently close together that the end of one gene may overlap the SD sequence or the coding sequence of the next gene. Eyre-Walker and Bulmer showed that there is a change in composition at the end of genes, which is consistent with selection against the formation of mRNA secondary structures around the SD sequence [23]. Eyre-Walker also showed that the strength and location of the SD sequence do not vary significantly because of the close proximity of the prokaryote genes [24]. It seems, therefore, that the spacing lengths and stop codon usage adapt themselves to the presence of SD. Recently, in the fusellovirus SSV4, which has a compactly organized genome, a preference for the TGA stop codon has been found in genes that overlap their stop codon with the SD sequence of the next gene to form the pattern GGTGA as a SD motif [25]. In prokaryotes, it seems that some intergenic distances are less favored because of the presence of the SD sequence. A certain stop codon usage is therefore required to form the SD motifs, as has been described in viruses. In this paper we assess how the presence of the SD sequence affects the spacing lengths between adjacent genes and the stop codon usage.

## Results and Discussion

### Spacing lengths between prokaryote genes

**Figure 1:**
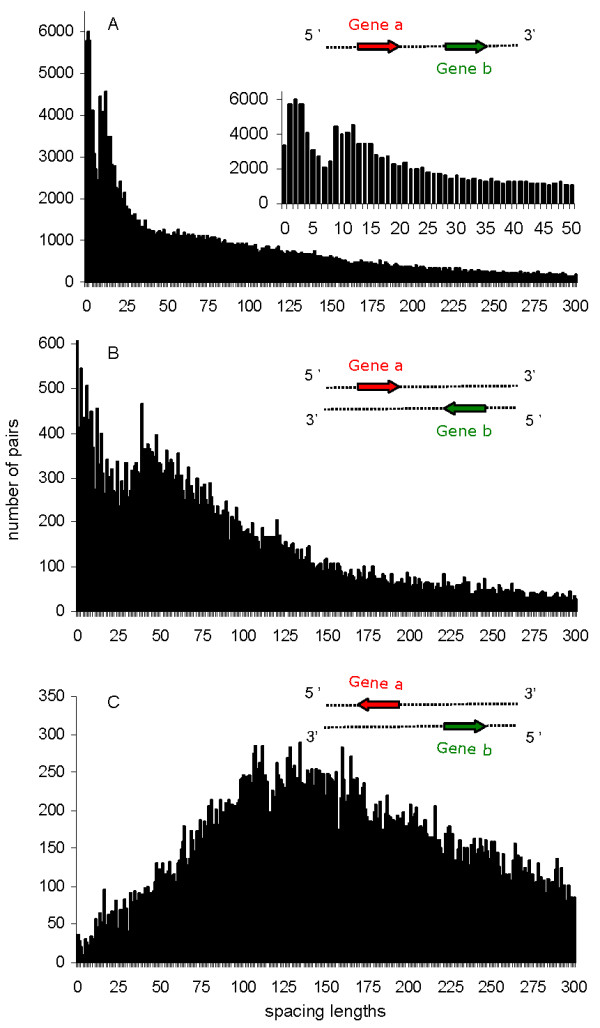
**Distribution of the spacing lengths**. Distribution of the spacing lengths between genes in co-directional (A), convergent (B) and divergent (C) orientations. A representation for each transcriptional orientation is shown. A distribution of the short spacing lengths between co-directional genes is shown in detail (A).

The distribution of the spacing lengths among the three gene orientations is different. This is probably due to the different gene structures found in each orientation (Figure 1). The co-directional number of pairs found in each spacing length decreases as the spacing length increases, though a long spacing length tail is observed (Figure 1A). The average spacing length among co-directional pairs is the lowest (163 bps) and the modal spacing length is 2 bps. The short modal spacing length reflects the fact that co-directional gene pairs tend to be grouped in operons and separated by short distances [3]. However, the long tail distances and the average spacing lengths of the co-directional pairs suggest that among the prokaryote genomes there is also a small minority of co-directional pairs that may be non-operonic. We have noticed that some prokaryote genomes have long intergenic regions that may be the result of pseudogenes accumulated in prokaryote genomes undergoing processes such as niche change, host specialization or weak selection strength [26]. The longest spacing lengths are found in *Mycobacterium leprae *and in the *Rickettsia *genomes, which appear to be in a process of extensive genome degradation via pseudogenization [27].

The convergent number of pairs in each spacing length decreases as the spacing lengths increase, as happens in co-directional spacing lengths, but the tail is much longer for long distances (Figure 1B). However, there is a noticeable increase in the number of pairs at a spacing length of around 30 bps that may be related to the presence of terminators, but this increase is not addressed in this paper. Although the modal spacing length is similar to the co-directional distribution (0 bps), the higher mean of the convergent spacing lengths (195 bps) indicates that these spacing lengths tend to be longer than co-directional ones, probably because the convergent gene pairs are basically inter-operonics. In contrast, the distribution of the divergent spacing lengths is totally different. The divergent number of pairs increases gradually to around 100 bps and remains high at around 175 bps before decreasing gradually with a long tail for long distances between genes (Figure 1C). The divergent distribution shows the highest mean of the spacing lengths (273 bps) and a modal spacing length of 135 bps. These results indicate that the divergent gene pairs, like the convergent ones, are basically inter-operonics. However, they require a longer space between them than the convergent and co-directional pairs, probably because of the need to accommodate several upstream regulatory signals for both genes of the pair [28]. Maintaining the upstream regulatory signals therefore seems to constrain the compression of the DNA more than the operon structures or termination signals. Note also that the convergent spacing lengths appeared to follow a phase bias, at least among the short ones (up to 30 bps) (Figure 1B). This phase bias is the result of the continuous creation and elimination of overlaps among the closely spaced genes. This uneven distribution of small separation distances is caused by the non-uniform distribution of reverse-complement stop codons [29]. Phase 0 (x = 0, 3, 6, 9, ...), which is prevalent, has the highest concentration of stop codons. Neither co-directional nor divergent pairs show any phase bias.

### Insights into the short spacers between co-directional genes

**Figure 2:**
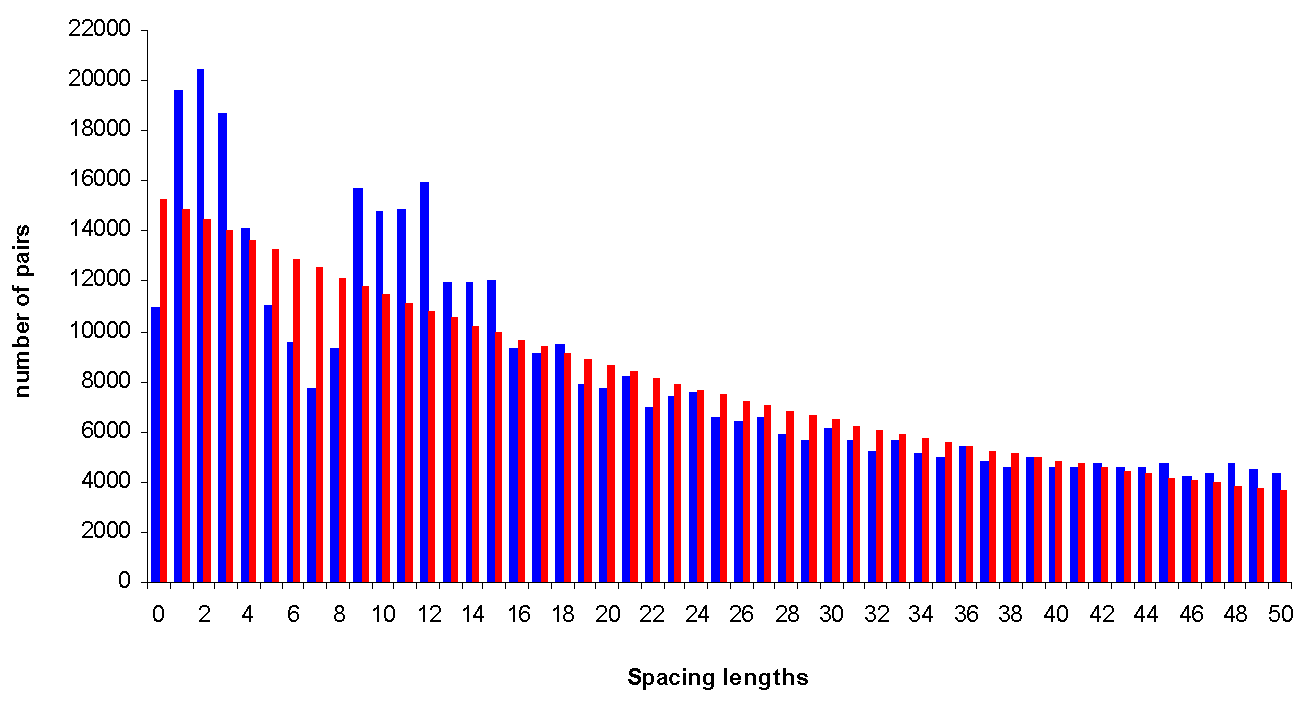
**Observed vs. expected co-directional spacing lengths**. Red bars show the expected number of gene pairs separated by each spacing length. Blue bars show the observed number of gene pairs separated by each spacing length up to 50 bps among the co-directional spacing lengths.

We focused on the fluctuations within the short co-directional spacing lengths (up to 15 bps). Apparently, there is a decrease in the co-directional gene pairs separated by spacing lengths of around 5 to 8 bps (Figure 1A). To confirm such fluctuations we fit a smoothed decay function of the form

to the observed distribution of co-directional spacing lengths x (Figure 2). We obtained values a = 15,301.4 and b = -0.03 for the parameters by fitting a least-squares regression line to the logarithm of the values in the histogram of observed spacing lengths over the range x = 0...50 (R^2 ^= 0.8496). We used a function of this form because we expected an exponential drop-off due to the large number of short spacing lengths between co-directional genes and because of the selective pressure to reduce the non-coding DNA content [1,2]. The combination of these facts may contribute to an exponential distribution of the spacing lengths, with a peak around short spacers and an exponential decay. By comparing the expected number of pairs with the observed number of pairs in every spacing length, we found three areas that provide relevant biological information (Figure 2). The intergenic lengths from 1 to 4 bps and from 9 to 15 bps showed an overrepresentation of the number of pairs but from 5 to 8 bps the number of pairs dropped off. Beyond 15 bps, the number of pairs observed for every spacing length was more similar to the expected number. To investigate these fluctuations, we studied the three areas mentioned above and included two others for the purpose of comparison. These extra areas were the spacing lengths beyond 15 bps and the whole spacing lengths. Despite the tendency towards a reduction in non-coding DNA, genomes must maintain a spacer between co-directional genes that should be long enough to accommodate the SD sequence. The presence of a SD sequence may therefore influence the length of the spacers because it is usually located between the upstream gene stop codon and the downstream gene start codon of the pair, while a proper distance between the SD sequence and the downstream gene start codon is maintained [12].

### SD presence within the prokaryote genomes

To detect the presence and location of the SD sequence we used an approach based on free energy calculations (described in the methods section; also see [13]). The genomes that have more genes with a predicted SD sequence belong to the Firmicutes phylum, especially the Bacillales species, and *Listeria innocua *Clip11262 is the genome that has the most genes with a predicted SD sequence (92.11% of its genes). The genome that has the fewest genes with a predicted SD sequence is the Bacteroidetes species *Flavobacterium psychrophilum *JIP02/86 (4.27% of its genes) (Additional File 1). As other authors have pointed out [14], the fact that the number of genes with a SD sequence varies from 4.27% to 92.11% implies that the populations of leaderless genes or genes without a SD sequence are really significant. Moreover, 249 of the 530 prokaryote genomes analyzed here have less than 50% of their genes with a predicted SD sequence and around 32 have fewer than 20% of their genes with a predicted SD sequence (Additional File 1). This could indicate that some prokaryote genomes use alternative translation initiation processes to translate their genes. Finding alternative processes to the SD-guided process is a still-unresolved issue.

Genomes that have a large number of genes with a predicted SD sequence seem to concentrate such a regulatory motif in a distance that ranges from 4 to 11 bps before the start codon (Additional File 1). This range is slightly different from the one previously defined (from 7 to 12 bps) [12,14,22]. The difference is due to the fact that we are calculating the distance from the base that binds the 5'A of the 16S rRNA tail sequence 3'-CCUCCA-5' to the first base of the start codon [13]. Other authors calculate the difference from the core of the SD sequence to the start codon and obtain longer distances. For genomes with very low percentages of SD presence, the distance range between the SD and the start codon is more scattered. Also, as the percentage of genes with a SD sequence decreases, the number of potential *mispredicted start codons or downstream start codon reflections *(see Methods section) increases, though the correlation is low (R^2 ^= 0.255). It appears, therefore, that prokaryote genomes, whose translation initiation process is mainly guided by the SD motif that binds the ribosome, conserve an optimal space between the SD motif and the start codon that can vary slightly depending on the species.

### Location of the SD motif within the short intergenic spacers between co-directional genes

**Figure 3:**
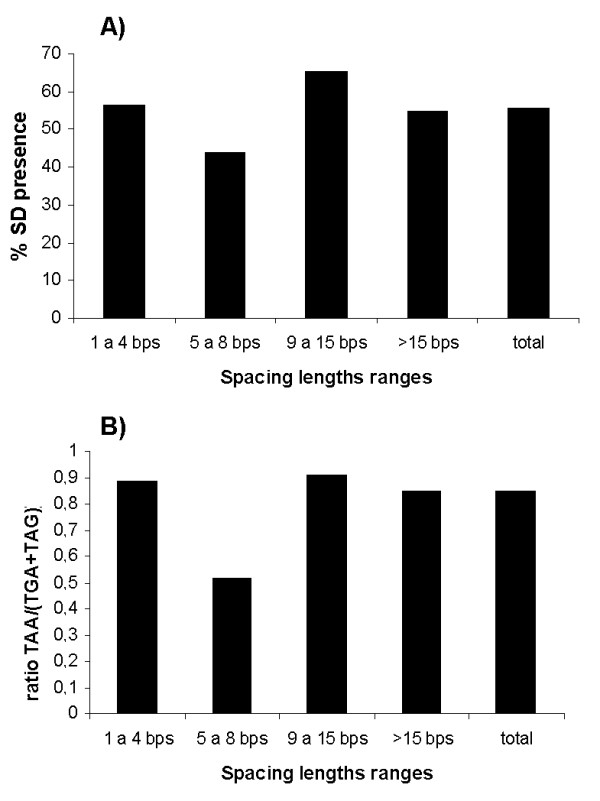
**SD presence and stop codon usage among the co-directional gene pairs**. Percentage of genes with a predicted SD sequence divided by the number of genes without a predicted SD sequence (A) and ratio of TAA usage to TGA and TAG usage as a stop codon (B) among the spacing lengths analyzed. The bars correspond to the ranges of spacing length studied and defined in the text.

We studied the presence or absence of the SD sequence within the co-directional gene pairs separated by the various ranges of spacing length. The set containing all the gene pairs has a 55.64% SD presence (Figure 3A). The percentage is similar for the set of gene pairs separated by spacers over 15 bps (54.93%) (Figure 3A). Although the translation in prokaryotes is mainly guided by the SD sequence that can bind the ribosome [12], it seems that the number of genes with SD sequence is only slightly higher than those without SD sequence. These results support the idea that non-SD-led genes are as common as SD-led genes [14]. The SD sequence of gene pairs separated from 1 to 4 bps should be along the end of the coding sequence of the previous gene. Although, as Eyre-Walker found [24], this constrains the 3'-end of the upstream gene, we found a higher number of genes with a predicted SD sequence and separated from the previous one by a spacing length from 1 to 4 bps. In this spacing length range, the percentage of genes with a SD sequence is slightly higher (56.33%) than in the total gene set (Figure 3A). Among the spacing lengths ranging from 9 to 15 bps we found the highest percentage of genes with a predicted SD sequence (65.32%) (Figure 3A). This may indicate that among the spacing lengths ranging from 9 to 15 bps, we generally find the optimal distances among genes that allow a least constrained accommodation of the SD motif, which is usually 4-11 nucleotides from the start codon in prokaryotes (Additional File 1). On the other hand, among spacing lengths between 5 and 8 bps, we observed a decrease in the number of genes with a predicted SD sequence (43.97%) (Figure 3A). It appears, therefore, that genes with a SD sequence may preferentially have an intergenic distance to the previous gene shorter than 5 bps (with the SD sequence overlapping the upstream coding sequence) or longer than 8 bps (with the SD sequence well accommodated within the intergenic region). Among the intergenic spacers from 5 to 8 bps the SD motif of a gene overlaps the previous stop codon, thus constraining the spacing length and stop codon usage of the previous gene. For longer intergenic distances, spacing lengths below 40 bps between co-directional genes are associated with genes belonging to the same operon [30] and the SD presence is significantly higher in genes within operons [12]. In fact, we found that co-directional genes separated by intergenic spacers below 44 bps have a higher percentage of genes with a predicted SD sequence than genes without one, while for intergenic distances above 44 bps the percentages are similar (data not shown).

The optimal distance range between the SD sequence and the start codon can vary depending on the genome (Additional File 1 and [12]) and this could vary the frequency of the short intergenic spacers. The distribution of distances between co-directional genes also varies among the taxonomical groups (Additional File 2). For each phylum, some distances are more frequent and others are less frequent within the short distances between co-directional genes. The less frequent distances are: from 5 to 8 bps in Acidobacteria, Aquificae and Proteobacteria; from 6 to 8 bps in Actinobacteria, Deinococcus-Thermus and Euryarcheota; from 7 to 8 bps in Chloroflexi, Crenarchaeota, Cyanobacteria, Chlorobi and Spirochaetes; from 7 to 9 bps in Bacteroidetes; from 9 to 11 bps in Synergistetes; from 5 to 10 bps in Firmicutes; from 6 to 10 bps in Thermotogae; and from 6 to 7 bps in Chlamydiae. There is no clear range in Planctomycetes. In general, it seems that the least frequent distances between co-directional genes are around 7 bps among all phylums except Planctomycetes and Synergistetes. The most represented phylums -- Proteobacteria, Euryarchaeota, Firmicutes and Actinobacteria (Additional File 2) -- were selected to perform an ANOVA analysis in order to determine whether the taxonomy and distances between co-directional genes could affect the presence of the SD sequence (Additional File 3). We observed significant differences among the distances (P = 0.040) and phylums analyzed (P < 0.0005). We performed a Post Hoc test to determine among which groups such differences exist (Additional File 3) and observed significant differences from 5 to 8 bps and from 9 to 15 bps (P = 0.032; Tukey test). This could be because the intergenic spacers from 9 to 15 bps accommodate the SD sequence best, while the presence of a SD sequence makes spacers from 5 to 8 bps less frequent. With regard to the phylums we observed significant differences between Firmicutes and Actinobacteria (P = 0.001; Tukey test), between Firmicutes and Proteobacteria (P < 0.0005, Tukey test), and between Firmicutes and Euryarchaeota (P = 0.011, Tukey test). This could be because Firmicutes tend to have a longer range of less-favored distances between co-directional genes (from 5 to 10 bps). The presence of underrepresented short distances and overrepresented short distances seems to be general among prokaryote genomes, though the less frequent and more frequent short distances vary across the species.

### Adaptation of the stop codon usage and spacing lengths to the presence of the SD sequence

The termination codons in the standard genetic code are TAA, TGA and TAG. TAA is the preferred stop codon in prokaryotes because of the greater availability of TAA-cognate release factor(s) or lower levels of translational readthrough [31]. It has been reported that TAA is used in preference to TGA, which itself is used in preference to TAG [31]. The stop codons TGA and TAG may be used when they have an additional function to coding for a stop signal [24]. For example, the TGA stop codon is used in one of the extremely common overlaps found in prokaryotes, the co-directional overlap of 4 bps, which includes the TGA stop codon of an upstream gene and the start codon of a downstream gene (ATG, GTG or TTG) [32,33]. The ratio of the TAA stop codon to the sum of the TGA and TAG stop codons in each range of spacing lengths is close to 1 (0.85-0.91) except for genes separated from 5 to 8 bps (Figure 3B), whose ratio is 0.52. It appears, therefore, that an upstream gene separated by 5-8 bps to the next one more frequently uses TGA or TAG as the stop codon (Figure 3B). Also, the SD presence of the downstream gene decreases within these spacing lengths (Figure 3A). This adaptation of a gene's stop codon usage could be caused by the SD sequence of the next gene overlapping its stop codon. The stop codons TGA and TAG may fit more easily within the SD motif. In a virus, whose genome is highly compacted, it was recently reported that overlaps of a stop codon and the SD sequence resulted in a common motif GGTGA. This is a clear adaptation of the upstream gene stop codon usage, which becomes part of the SD motif and maintains its function as stop codon.

**Figure 4:**
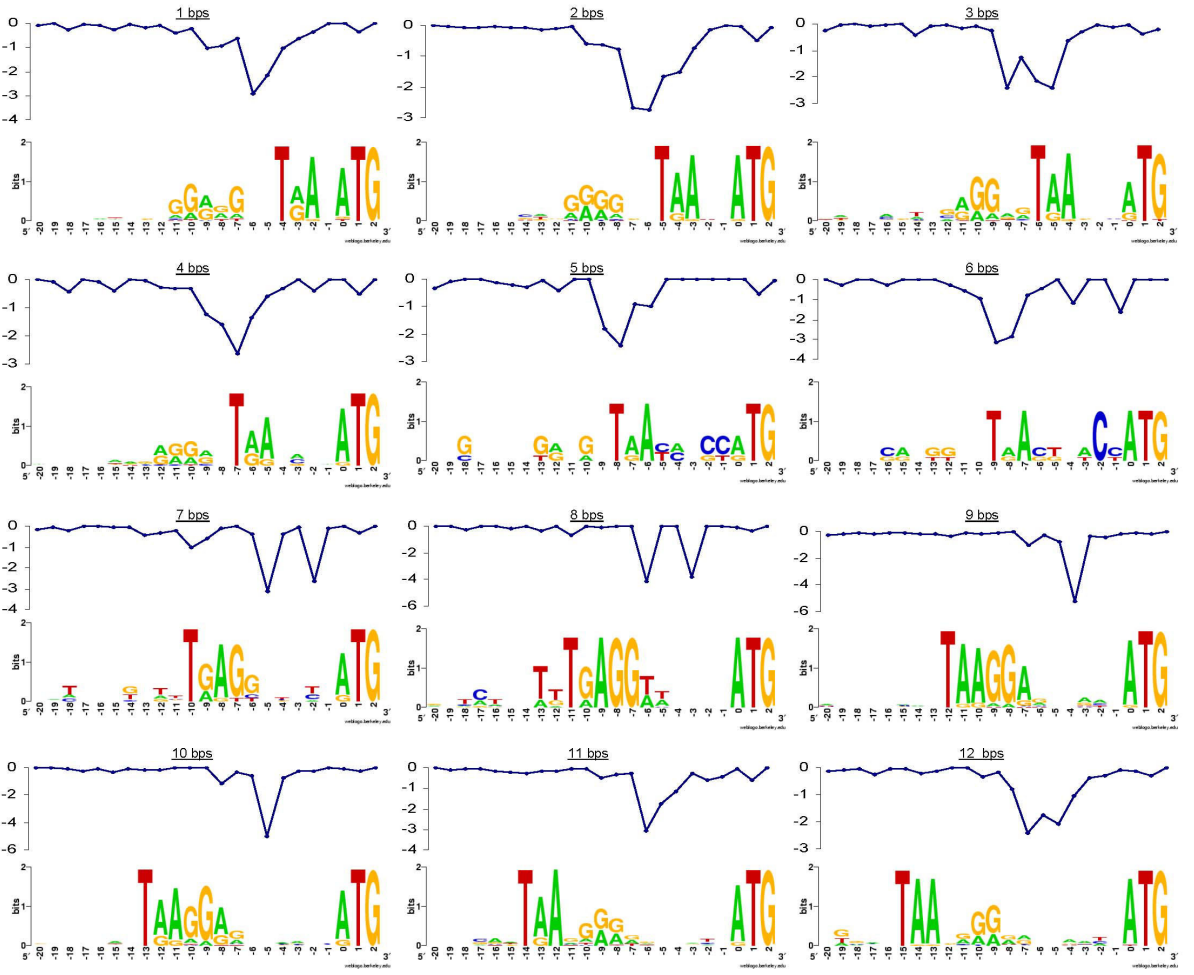
**Sequence logos for *E. coli *K12**. Sequence logos of the nucleotides between positions -20 and 2 of the genes with a spacer from 1 to 12 bps long. For each position, the sequence logo shows the amount of information content and the frequency of nucleotides. The blank positions mean there is no information content. Those with information content contain a stack of nucleotides. The size of the nucleotide character is proportional to its frequency at that position. Each sequence logo has an average ΔG° value ranging from -20 to 2 bps of the genes separated by each of the spacers analyzed. The higher the ΔG° value, the stronger the binding between the 16S rRNAs and the mRNAs. The drops in ΔG° values indicate where the 5'A of the 16S rRNA tail (3'-CCUCCA-5') can bind the SD sequence. These drops are before the regions with high frequencies of As and Gs.

To study the possible adaptation of the stop codon usage and spacing lengths to the SD presence among the short spacers between co-directional genes, we built sequence logos for the intergenic regions of *Escherichia coli *K12 from 1 to 12 bps (Figure 4). In *E. coli *K12 the number of adjacent genes separated by spacing lengths from 1 to 4 bps and from 9 to 13 bps is overrepresented, while from 5 to 8 bps it is underrepresented. From 1 to 4 bps we observed a high proportion of As and Gs before the upstream stop codon, which may indicate the presence of a SD sequence along the end of the upstream gene coding sequence (Figure 4). From 2 to -20, a drop in the ΔG° value is observed before these regions with a high frequency of As and Gs. The stop codon usage is biased to the TAA use and the proportion of genes with a predicted SD sequence and without a predicted SD sequence is higher than 1 in each spacer (Table 1). From 5 to 6 bps we found few genes with a SD sequence to build the logo (4 and 3, respectively). In fact, there are more genes without a predicted SD sequence than with a predicted SD sequence in genes separated by these spacing lengths (Table 1). Therefore, the 5 and the 6 bps distances are the ones most compromised by the presence of the SD sequence. From 7 to 8 bps the high frequency of As and Gs suggests that the SD sequence is overlapping the downstream gene stop codon and there is a drop in ΔG° value just before the stop codon (from 2 to -20). Interestingly, the SD sequence seems to adopt the TGAGG pattern when the *E. coli *K12 genes are separated by 7 or 8 bps (Figure 4). From 9 to 12 bps the high frequency of As and Gs is between the upstream gene stop codon and the downstream gene start codon. The drop in ΔG° value is around the middle of the intergenic region and the TAA stop codon is used in preference. In fact, this stop codon could bind well with the end bases of the *E. coli *K12 16S rRNA tail (3'-AUU-5'), especially for a length of 9 and 10 bps (Figure 4).

**Table 1:** Co-directional genes with and without a SD sequence separated by short intergenic spacers in *E. coli *K12

Spacing lengths (bps)	Genes with a predicted SD sequence	Genes without a predicted SD sequence
1	22	17

2	28	22

3	31	16

4	14	13

5	4	8

6	3	10

7	14	11

8	10	6

9	48	8

10	48	6

11	39	9

12	30	10

Number of genes with a SD sequence and without a SD sequence in each spacing length from 1 to 12 bps in the *E. coli *genome.

When the SD sequence overlaps the upstream coding sequence or stop codon, its strength and relative distance to the downstream start codon do not vary significantly (see Figure 4, Additional File 1 and [24]). However, the presence of a SD sequence may make the intergenic lengths from 5 to 8 bps less frequent and cause an adaptation of the stop codon usage. In *E. coli *K12 this adaptation is reflected in the prevalence of TGA usage forming the **TGA**GG pattern for co-directional genes separated by 7 or 8 bps. However, the adaptation could be slightly different depending on the prokaryote species. For instance, in the *B. subtilis *genome with less frequent intergenic lengths from 7 to 10 bps, the co-directional genes separated by 10 bps use TAG rather than TGA as the stop codon. This results in the SD motif **TAG**GAGG (Additional File 4A). There is a drop in the average of the free energy values just before this pattern, which indicates a strong binding of this SD pattern with the fragment of the *B. subtilis *16S rRNA tail, 3'-UUCCUCC-5' (Additional file 4A). On the other hand, for the archaea *Thermococcus kodakarensis *the SD pattern is GG**TGA**, which is described as a common SD pattern among archaeas [34]. In this pattern, the stop codon TGA is involved in the SD pattern, which could be responsible for binding the fragment of the *T. kodakarensis *16S rRNA tail, 3'-CCACU-5' (Additional File 4B). In the archaea, the drop in the average of the free energy values is on the T of the TGA stop codon (Additional File 4B).

Two mechanisms may cause the SD sequence to overlap with a TGA or TAG stop codon. The first involves the deletion of a portion of an intergenic sequence followed by a mutation at the upstream stop codon that changes the most frequent TAA stop codon to TGA or TAG. The second involves a single step -- the deletion of a portion of an intergenic sequence when the stop codon of the upstream gene is already a TGA or TAG. This second mechanism is the more parsimonious of the two.

## Conclusion

Among co-directional genes, the intergenic spacers from 5 to 8 bps are the least frequent because the SD sequence overlaps the previous stop codon. In this case the strength and relative distance of the SD sequence to the downstream start codon do not vary significantly. However, the SD sequence may be slightly different from the canonical SD motif (GGAGG). Not all stop codons may overlap with a SD sequence. The least frequent stop codons (TGA and TAG) fit better within a SD sequence. Therefore, a variation of the stop codon usage takes place when the length of the next spacer is from 5 to 8 bps and this spacer contains a SD motif. Our results introduce new elements in the discussion of which factors other than the pressure of genome compaction may affect the length of intergenic regions in prokaryotes.

## Methods

### Data retrieval and study of the distribution of spacing lengths

The complete genome sequences of 530 chromosomes from prokaryotes were downloaded from the NCBI ftp site ftp://ftp.ncbi.nlm.nih.gov/genomes/. Perl scripting was used to extract the overlaps and the spacers between adjacent genes. However, we excluded the overlapping genes and only considered the adjacent genes separated by 0 or more bps. For these genes, the spacing length is defined as the distance between the end of the upstream gene and the start of the downstream gene. Although we calculated all the spacing lengths between the genes contained in the 530 chromosomes from the prokaryotes analyzed, we focused our study on the co-directional spacing lengths.

Unfortunately, in prokaryotes all analyses of intergenic regions are hampered by annotation errors such as incorrect initiation codon prediction, falsely predicted genes and frameshifts [35-38]. To check whether incorrect initiation codon predictions affect our conclusions, we analyzed the distribution of distances between co-directional genes calculated with the NCBI annotations and those calculated with the annotations refined by triTISA [39], a post-processor program for refining the annotations of translation initiation site. We compared the two distributions (Additional File 5) using paired samples T-test and found significant differences (P < 0.0005). However, both groups followed the same tendency and there was a strong linear correlation (R^2 ^= 0.990) (Additional File 5). For both distributions, the larger the spacing lengths the fewer the number of pairs, though there are more frequent and less frequent distances within the short spacers between co-directional genes (Figure 1A and Additional File 5). We therefore concluded that, although incorrect gene annotations exist, they do not influence our results or conclusions. It is worth commenting that, in general, we observed slightly longer intergenic distances among the refined set, which it is pointing out the tendency of RefSeq to predict as gene the longest open reading frame [40].

### Stop codon usage analysis

As we studied the co-directional spacing lengths we only considered pairs of genes with an orientation (->->) or (<-<-). When we took into account the DNA direction from 5' to 3', in the case of orientation (->->) we looked at the stop codon of the upstream gene. When we took into account the DNA direction from 3' to 5', in the case of orientation (<-<-) we looked at the stop codon of the downstream gene. The region that involves the upstream gene stop codon, the possible downstream SD motif and the downstream gene start codon (from -20 to 2) was represented by WebLogo [41] in *E. coli *K12 spacing lengths from 1 to 12 bps (Figure 4), in *B. subtilis *spacing length 10 bps, and in *T. kodakarensis *spacing length 6 bps (Additional File 5). The numbers of genes with a predicted SD sequence considered for building each WebLogo in *E. coli *K12 are shown in Table 1, while for *B. subtilis *and *T. kodakarensis *they are shown in the legend of Additional File 5.

### Location of the SD motif

Since the SD sequence was discovered and characterized [8], two approaches have been used to identify and locate the SD motif in prokaryotes. These approaches are based either on sequence similarity or free energy calculations. In this paper we used the method of Starmer and co-workers, which is based on free energy calculations [13]. We chose this method because it is based on thermodynamic considerations of the 30S binding to the mRNA and overcomes the limitations of sequence analysis [13]. We obtained the 16S rRNAs from the NCBI ftp site ftp://ftp.ncbi.nlm.nih.gov/genomes/. For each 16S rRNA sequence of each organism we looked in the 5' direction for the first instance of the three letter motif, 5'-GAT-3', which was consistently found at the 5' end of the tails of the 16S rRNAs with known structures [42]. The location of this motif was used to define, up to the end of the 3' tail, the 16S rRNA tail of each organism (see Additional File 1). For species with two or more copies of the 16S rRNA gene, we calculated the consensus sequence of all the tails. If the tails did not follow a consensus, we used the majority of the 16S rRNA gene tails. Finally, all the 16S rRNA tails of the 530 organisms were examined manually, especially the short ones because we detected that most of the genomes with short 16S rRNA tails have their end of the 16S rRNA badly annotated. This finding agrees with a recent paper that states that some annotations of 16S rRNAs in RefSeq are questionable and should be improved [43]. We corrected these bad annotations of the 16S rRNA tails by comparing the 16S rRNAs of related species with BLAST [44].

The scripts to calculate the free energies of the 16S rRNA tail binding with the mRNA were downloaded from http://sourceforge.net/projects/free2bind/ and included in our Perl scripts. We located the SD sequence from the position of the lowest ΔG° value calculated from 35 bps upstream to the initiation codon to 35 bps downstream from the initiation codon. The gene was assumed not to have a SD sequence if ΔG° > -3.4535 Kcal/mol. The threshold we used is based on the work of Ma and co-workers [12]. To pinpoint the exact SD position we used the relative spacing parameter [13], which means that we calculated the distance between the first residue of the start codon and the 5' A of the rRNA sequence 5'-ACCUCC-3' in the positions around the start codon. If the SD motif is located before the start codon the relative spacing is negative; if the SD motif is located after the start codon the relative spacing is positive.

### Classifying the SD motif signal

**Figure 5:**
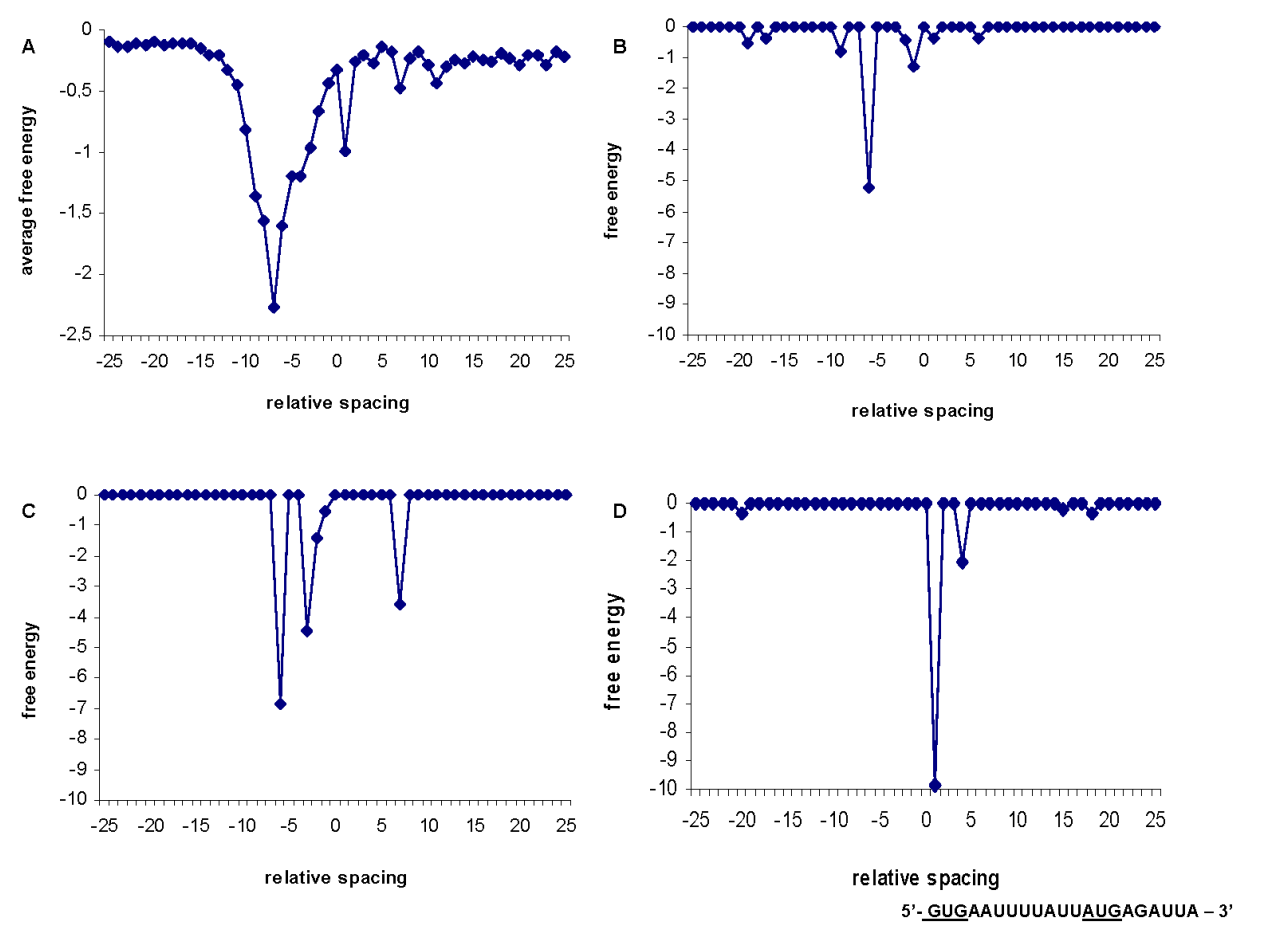
**ΔG° values in the translation initiation region for the *C. tetani *E88 genes**. For all the *C. tetani *E88 genes we calculated the average of ΔG° values in the translation initiation region for each relative spacing position (A). A dramatic drop in the ΔG° values from 5 to 11 nucleotides before the start codon indicates presence of SD sequence. The sudden drop in the ΔG° value immediately after the first base in the start codon may indicate potentially wrong SD predictions, while the sudden drop at, for instance, 7 bps may indicate downstream start codon reflections. A drop in the ΔG° values at 6 bps to the start codon of the CTC00136 gene indicates that it is a gene with an upstream SD sequence (B). The gene CTC00983 shows three drops in ΔG° value (C). The drop at 6 bps to the start codon falls within the optimal distance between the SD sequence and the start codon (from 5 to 11 bps), while the drop at 3 bps falls out of this optimal distance. Looking downstream of the start codon the drop in ΔG° value falls at 7 bps after the first base of the start codon, which may mean that there is a start codon reflecting a SD sequence around this position. A dramatic drop in ΔG° value is observed 1 bps after the first base of the GTG start codon of the gene CTC00194 (D). This drop is indicating a mispredicted start codon (GTG underlined in the sequence) and as it can be observed in the downstream sequence, which is denoted below the graph, this gene has an alternative start codon (ATG underlined in the sequence) only 4 codons downstream of the mispredicted one.

Taking into account the various drops in ΔG° value and the most frequent distance between the upstream SD sequence and the start codon in each genome, we divided the various SD motif signals into four groups. The PairWise Neighbours database helped us to differ the different types of SD motif signals [45]. Figure 5 shows the average ΔG° values within the translation initiation region for *Clostridium tetani *E88 genome (Figure 5A) and the ΔG° values within the translation initiation region in three different genes of this genome. We consider that a gene has an *upstream SD sequence *if it has at least one clear drop in ΔG° value within the most frequent distance range (from 5 to 11 bps in *C. tetani *E88; see Additional File 1) between the SD sequence and the start codon (Figure 5B). If a gene has drops in ΔG° value upstream and downstream to the start codon and one of these falls within the most frequent distance range between the SD sequence and the start codon for the genome, we consider that the gene has *SD sequence and a downstream start codon reflection *(Figure 5C). A downstream start codon reflection means two possible events. One of these is unspecific binding between the 16S rRNA tail and the mRNA. For example, the start codon GUG is very close to the central core of a SD sequence, GGUGG. The U of the 16S rRNA tail CCUCC can also bind to A or U. Therefore, the GUG start codon, for example, may cause unspecific bindings. The other possible event is a SD sequence downstream from the predicted start codon. This would imply that the start codon was previously wrongly predicted and the real one is located downstream along the sequence. A sudden drop in ΔG° value at 1 bps from the beginning of the start codon (in the second nucleotide of the start codon) exposes a wrongly predicted start codon [13]. For example, the *C. tetani *E88 gene CTC00194 has a downstream prediction at 1 bps and the start codon is GTG, but the actual one should be the ATG codon located 4 codons downstream and the GTG one should be part of the SD sequence (Figure 5D). We called the genes with drops in ΔG° value only downstream a *mispredicted start codon *or *a downstream start codon reflection *(Figure 5D).

These genes with a predicted SD sequence downstream from the start codon usually have an overrepresentation of the GTG codon as the start codon (Table 2). The gene groups called *SD sequence and downstream start codon reflection *and *mispredicted start codon or downstream start codon reflection *show a percentage GTG of 62.7% and 65.4%, respectively (Table 2). In comparison with genes with a predicted upstream SD sequence (9.5%) or without a predicted SD sequence (11.2%), which tend to use more ATG as start codon (Table 2), these are high percentages.

**Table 2:** Start codon usage among the SD populations

	Start codon usage			
Start codons	*upstream SD sequence *(% genes)	*SD sequence and downstream start codon reflection *(% genes)	*mispredicted start codon or downstream start codon reflection *(% genes)	*without SD sequence *(% genes)
**AUG**	84.8%	33.8%	29.7%	79.8%
**GUG**	9.5%	62.7%	65.4%	11.2%
**UUG**	5.6%	3.4%	4.6%	8.7%
**other**	0.1%	0.1%	0.3%	0.3%

Percentages of start codon usage among genes with an *upstream SD sequence*, genes with a *SD sequence and downstream start codon reflection*, genes with a *mispredicted start codon or downstream start codon reflection*, and genes *without SD sequence*.

For our analysis purposes of SD presence, genes like those in Figures 5B and 5C were considered genes with a predicted SD sequence (*upstream SD sequence *and *SD sequence and a downstream start codon reflection *groups). Genes like those in Figure 5D (*mispredicted start codon or a downstream start codon reflection*) were not considered in our calculations and the genes without drops in ΔG° values (see the threshold for the ΔG° values in the previous subsection) in the translation initiation region were considered genes *without SD sequence*.

## Authors' contributions

AP performed the Perl Scripts to obtain the intergenic sequences and the genomic and SD information. AP also sorted all this information and built the WebLogos sequence. AP, SGV and AR helped to analyze and interpret the data. AP drafted the manuscript and SGV and AR critically reviewed it. Finally, all the authors read and approved the version for publication.

## Supplementary Material

Additional file 1 **Percentage of SD presence, preferred location for the SD motif, and 16S rRNA tail used of each prokaryote genome**. Excel file showing the percentage of genes in each SD populations (see Materials and Methods), the optimal distance between the beginning of the SD motif and the first base of the start codon and the 16S rRNA tail used to calculate the binding between the 16S rRNA and the mRNA for each prokaryote chromosome. Taxonomical information for each prokaryote genome is also given. The 530 prokaryote chromosomes are sorted by the percentage of genes with a predicted SD sequence. Click here for file

Additional file 2 **Distribution of the spacing lengths by phylums**. Excel file showing the distribution of the short spacing lengths up to 50 bps between the co-directional genes grouped by phylums. Click here for file

Additional file 3 **Statistical analysis of the SD presence**. Word file showing the results of the ANOVA analysis to check whether the taxonomy and the distances between co-directional genes can affect the presence of the SD sequence. Post Hoc tests (Tukey test) were performed in both factors to determine which levels have differences. Significant differences have a P value < 0.05 and are denoted in bold. Click here for file

Additional file 4 **Sequence logos for *B. subtilis *and *T. kodakaraensis***. Weblogos showing the SD pattern for *B. subtilis *when there is a distance between genes of 10 bps (9 genes considered) (A), and the SD pattern for *T. kodakaraensis *when there is a distance between genes of 6 bps (16 genes considered) (B). For each position (from -20 to 2 bps), the sequence logo shows the amount of information content and the frequency of nucleotides. The blank positions mean that there is no information content. Those with information content contain a stack of nucleotides. The size of the nucleotide character is proportional to its frequency at that position. Each sequence logo has the average of ΔG° values from -20 to 2 bps of the genes separated by each of the spacers analyzed. The higher the ΔG° value, the stronger the binding between the 16S rRNAs and the mRNAs. The drops in ΔG° values indicate where the 5'A of the 16S rRNA tail (3'-CCUCCA-5') can bind the SD sequence. These drops are before the SD patterns TAGGAGG in *B. subtilis *and on the T in the middle of the SD pattern GGTGA in *T. kodakaraensis*. Click here for file

Additional file 5 **Comparison of the distances between co-directional genes calculated with NCBI annotations and with triTISA**. Comparison of the distribution of the distances between co-directional genes calculated with the NCBI annotations and with the annotations refined with triTISA (A), and correlation between the intergenic distances calculated with the RefSeq annotations and with the annotations refined by the triTISA program (B). The figure shows the correlation coefficient and the linear equation. Click here for file
