# Supplementary material for: Adaptation of the short intergenic spacers between co-directional genes to the Shine-Dalgarno motif among prokaryote genomes
Source: BMC Genomics. 2009 Nov 18;10:537. doi: 10.1186/1471-2164-10-537 (PMC2784483; doi:10.1186/1471-2164-10-537)
Supplement: Additional file 3 — Statistical analysis of the SD presence. Word file showing the results of the ANOVA analysis to check whether the taxonomy and the distances between co-directional genes can affect the presence of the SD sequence. Post Hoc tests (Tukey test) were performed in both factors to determine which levels have differences. Significant differences have a P value < 0.05 and are denoted in bold. [file 1471-2164-10-537-S3.DOC]

**Univariate Analysis of Variance**

| **Between-Subjects Factors** | | | |
| --- | --- | --- | --- |
|  |  | Value Label | N |
| Distances | 1 | 1 to 4 bps | 4 |
|  | 2 | 5 to 8 bps | 4 |
|  | 3 | 9 to 15 bps | 4 |
|  | 4 | > 15 bps | 4 |
| Taxonomy | 1 | Firmicutes | 4 |
|  | 2 | Actinobacteria | 4 |
|  | 3 | Proteobacteria | 4 |
|  | 4 | Euryarchaeota | 4 |

| **Tests of Between-Subjects Effects** | | | | | |
| --- | --- | --- | --- | --- | --- |
| Dependent Variable:percentage SD presence | | | | | |
| Source | Type III Sum of Squares | df | Mean Square | F | Sig. |
| Corrected Model | 2670,242^a^ | 6 | 445,040 | 11,057 | ,001 |
| Intercept | 59570,651 | 1 | 59570,651 | 1480,035 | ,000 |
| Distances | 513,311 | 3 | 171,104 | 4,251 | **,040** |
| Taxonomy | 2156,931 | 3 | 718,977 | 17,863 | **,000** |
| Error | 362,245 | 9 | 40,249 |  |  |
| Total | 62603,139 | 16 |  |  |  |
| Corrected Total | 3032,488 | 15 |  |  |  |
| a. R Squared = ,881 (Adjusted R Squared = ,801) | | | | | |

**Post Hoc Tests**

***Distances***

| **Multiple Comparisons** | | | | | | |
| --- | --- | --- | --- | --- | --- | --- |
| percentage SD presence  Tukey HSD | | | | | | |
| (I) Distances | (J) Distances | Mean Difference (I-J) | Std. Error | Sig. | 95% Confidence Interval | |
|  |  |  |  |  | Lower Bound | Upper Bound |
| 1 to 4 bps | 5 to 8 bps | 1,10515071402353E1 | 4,486060994777587E0 | ,134 | -2,95307213775577E0 | 2,50560864182263E1 |
|  | 9 to 15 bps | -4,24331463430026E0 | 4,486060994777587E0 | ,782 | -1,82478939122913E1 | 9,76126464369077E0 |
|  | > 15 bps | 4,47143660544385E0 | 4,486060994777587E0 | ,755 | -9,53314267254718E0 | 1,84760158834349E1 |
| 5 to 8 bps | 1 to 4 bps | -1,10515071402353E1 | 4,486060994777587E0 | ,134 | -2,50560864182263E1 | 2,95307213775577E0 |
|  | 9 to 15 bps | -1,52948217745355E1 | 4,486060994777587E0 | **,032** | -2,92994010525265E1 | -1,29024249654449E0 |
|  | > 15 bps | -6,58007053479141E0 | 4,486060994777587E0 | ,493 | -2,05846498127824E1 | 7,42450874319962E0 |
| 9 to 15 bps | 1 to 4 bps | 4,24331463430026E0 | 4,486060994777587E0 | ,782 | -9,76126464369077E0 | 1,82478939122913E1 |
|  | 5 to 8 bps | 1,52948217745355E1 | 4,486060994777587E0 | **,032** | 1,29024249654449E0 | 2,92994010525265E1 |
|  | > 15 bps | 8,71475123974410E0 | 4,486060994777587E0 | ,277 | -5,28982803824692E0 | 2,27193305177351E1 |
| > 15 bps | 1 to 4 bps | -4,47143660544385E0 | 4,486060994777587E0 | ,755 | -1,84760158834349E1 | 9,53314267254718E0 |
|  | 5 to 8 bps | 6,58007053479141E0 | 4,486060994777587E0 | ,493 | -7,42450874319962E0 | 2,05846498127824E1 |
|  | 9 to 15 bps | -8,71475123974410E0 | 4,486060994777587E0 | ,277 | -2,27193305177351E1 | 5,28982803824692E0 |
| Based on observed means.  The error term is Mean Square(Error) = 40,249. | | | | | | |
| *. The mean difference is significant at the 0,05 level. | | | | | | |

**Homogeneous subsets**

| **percentage SD presence** | | | |
| --- | --- | --- | --- |
| Tukey HSD^a,,b^ | | | |
| Distances | N | Subset | |
|  |  | 1 | 2 |
| 5 to 8 bps | 4 | 5,27861490379642E1 |  |
| > 15 bps | 4 | 5,93662195727556E1 | 5,93662195727556E1 |
| 1 to 4 bps | 4 | 6,38376561781995E1 | 6,38376561781995E1 |
| 9 to 15 bps | 4 |  | 6,80809708124997E1 |
| Sig. |  | ,134 | ,277 |
| Means for groups in homogeneous subsets are displayed.  Based on observed means.  The error term is Mean Square(Error) = 40,249. | | | |
| a. Uses Harmonic Mean Sample Size = 4,000. | | | |
| b. Alpha = 0,05. | | | |

***Taxonomy***

| **Multiple Comparisons** | | | | | | |
| --- | --- | --- | --- | --- | --- | --- |
| percentage SD presence  Tukey HSD | | | | | | |
| (I) Taxonomy | (J) Taxonomy | Mean Difference (I-J) | Std. Error | Sig. | 95% Confidence Interval | |
|  |  |  |  |  | Lower Bound | Upper Bound |
| Firmicutes | Actinobacteria | 2,76016009542301E1 | 4,486060994777587E0 | **,001** | 1,35970216762391E1 | 4,16061802322211E1 |
|  | Proteobacteria | 2,92061395737540E1 | 4,486060994777587E0 | **,001** | 1,52015602957630E1 | 4,32107188517451E1 |
|  | Euryarchaeota | 1,85850808755340E1 | 4,486060994777587E0 | **,011** | 4,58050159754299E0 | 3,25896601535250E1 |
| Actinobacteria | Firmicutes | -2,76016009542301E1 | 4,486060994777587E0 | **,001** | -4,16061802322211E1 | -1,35970216762391E1 |
|  | Proteobacteria | 1,60453861952395E0 | 4,486060994777587E0 | ,983 | -1,24000406584671E1 | 1,56091178975150E1 |
|  | Euryarchaeota | -9,01652007869608E0 | 4,486060994777587E0 | ,253 | -2,30210993566871E1 | 4,98805919929495E0 |
| Proteobacteria | Firmicutes | -2,92061395737540E1 | 4,486060994777587E0 | **,001** | -4,32107188517451E1 | -1,52015602957630E1 |
|  | Actinobacteria | -1,60453861952395E0 | 4,486060994777587E0 | ,983 | -1,56091178975150E1 | 1,24000406584671E1 |
|  | Euryarchaeota | -1,06210586982200E1 | 4,486060994777587E0 | ,153 | -2,46256379762111E1 | 3,38352057977100E0 |
| Euryarchaeota | Firmicutes | -1,85850808755340E1 | 4,486060994777587E0 | **,011** | -3,25896601535250E1 | -4,58050159754299E0 |
|  | Actinobacteria | 9,01652007869608E0 | 4,486060994777587E0 | ,253 | -4,98805919929495E0 | 2,30210993566871E1 |
|  | Proteobacteria | 1,06210586982200E1 | 4,486060994777587E0 | ,153 | -3,38352057977100E0 | 2,46256379762111E1 |
| Based on observed means.  The error term is Mean Square(Error) = 40,249. | | | | | | |
| *. The mean difference is significant at the 0,05 level. | | | | | | |

**Homogenous subsets**

| **percentage SD presence** | | | |
| --- | --- | --- | --- |
| Tukey HSD^a,,b^ | | | |
| Taxonomy | N | Subset | |
|  |  | 1 | 2 |
| Proteobacteria | 4 | 5,06598146774802E1 |  |
| Actinobacteria | 4 | 5,22643532970042E1 |  |
| Euryarchaeota | 4 | 6,12808733757003E1 |  |
| Firmicutes | 4 |  | 7,98659542512343E1 |
| Sig. |  | ,153 | 1,000 |
| Means for groups in homogeneous subsets are displayed.  Based on observed means.  The error term is Mean Square(Error) = 40,249. | | | |
| a. Uses Harmonic Mean Sample Size = 4,000. | | | |
| b. Alpha = 0,05. | | | |
