# Supplementary figures and images for: Adaptation of the short intergenic spacers between co-directional genes to the Shine-Dalgarno motif among prokaryote genomes
Source: BMC Genomics. 2009 Nov 18;10:537. doi: 10.1186/1471-2164-10-537 (PMC2784483; doi:10.1186/1471-2164-10-537)

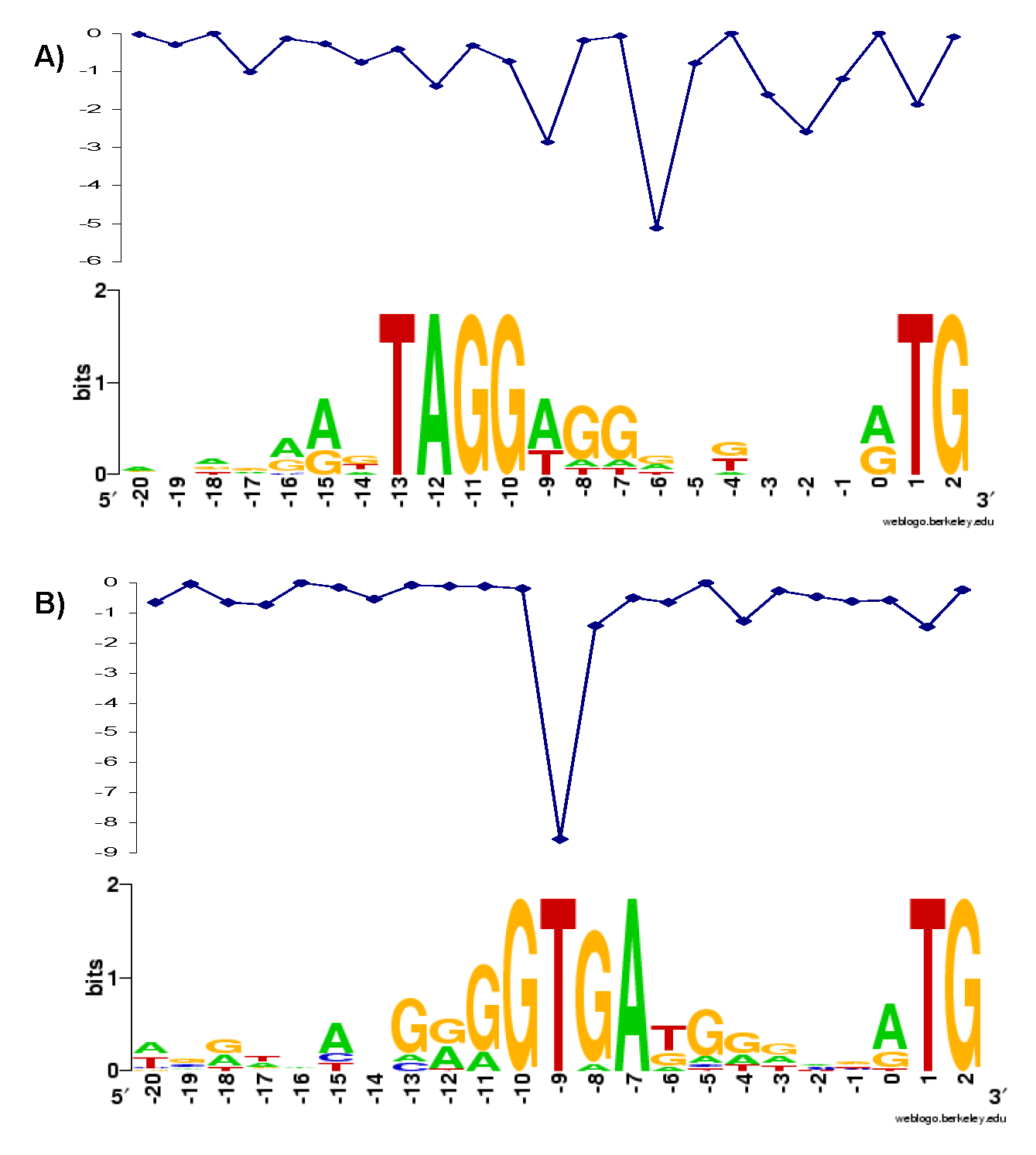

Supplement: Additional file 4 — Sequence logos for B. subtilis and T. kodakaraensis. Weblogos showing the SD pattern for B. subtilis when there is a distance between genes of 10 bps (9 genes considered) (A), and the SD pattern for T. kodakaraensis when there is a distance between genes of 6 bps (16 genes considered) (B). For each position (from -20 to 2 bps), the sequence logo shows the amount of information content and the frequency of nucleotides. The blank positions mean that there is no information content. Those with information content contain a stack of nucleotides. The size of the nucleotide character is proportional to its frequency at that position. Each sequence logo has the average of ΔG° values from -20 to 2 bps of the genes separated by each of the spacers analyzed. The higher the ΔG° value, the stronger the binding between the 16S rRNAs and the mRNAs. The drops in ΔG° values indicate where the 5'A of the 16S rRNA tail (3'-CCUCCA-5') can bind the SD sequence. These drops are before the SD patterns TAGGAGG in B. subtilis and on the T in the middle of the SD pattern GGTGA in T. kodakaraensis. [file 1471-2164-10-537-S4.TIFF]

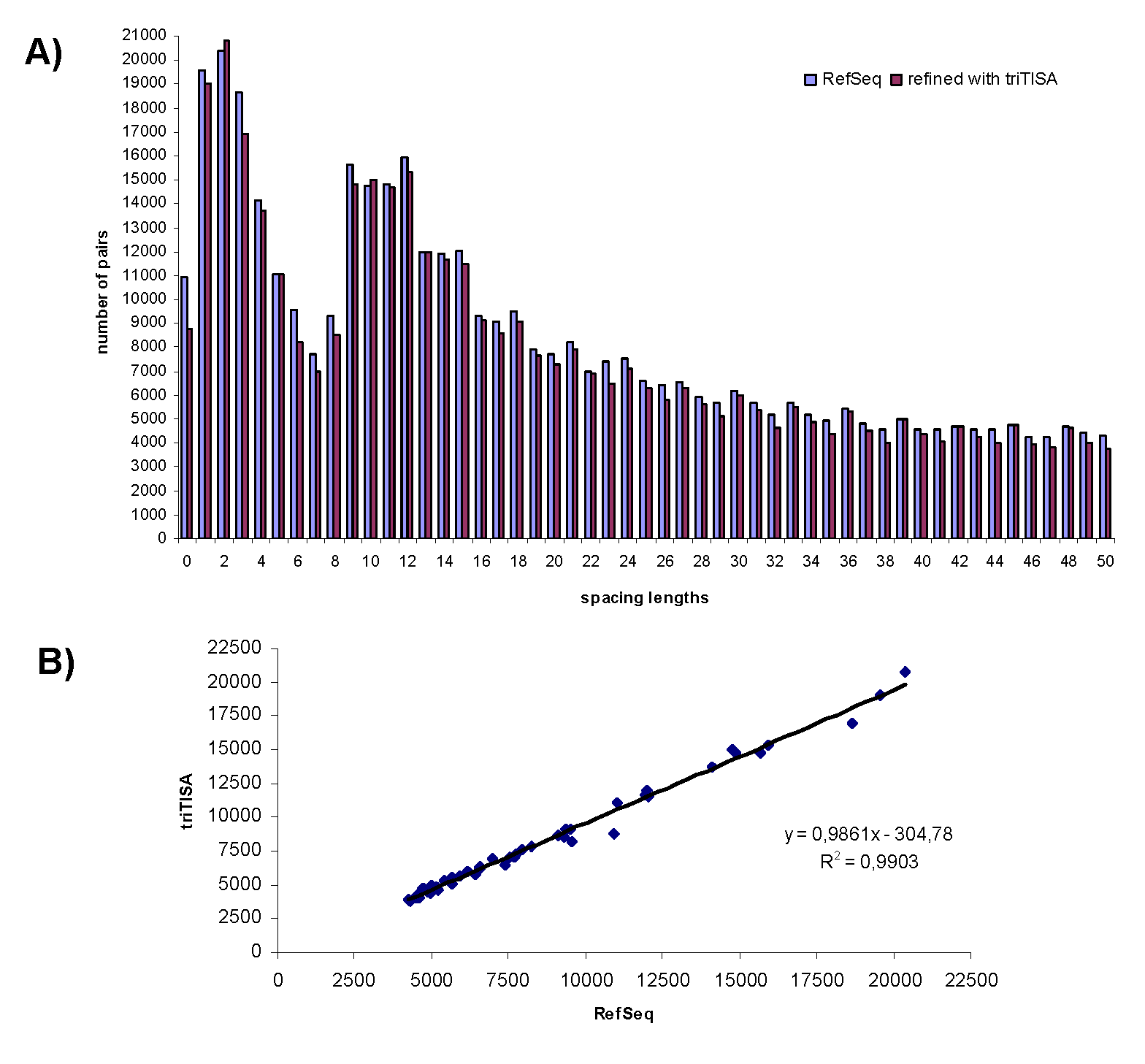

Supplement: Additional file 5 — Comparison of the distances between co-directional genes calculated with NCBI annotations and with triTISA. Comparison of the distribution of the distances between co-directional genes calculated with the NCBI annotations and with the annotations refined with triTISA (A), and correlation between the intergenic distances calculated with the RefSeq annotations and with the annotations refined by the triTISA program (B). The figure shows the correlation coefficient and the linear equation. [file 1471-2164-10-537-S5.TIFF]
